# Process optimization of spray‐dried Moldavian balm (*Dracocephalum moldavica* L.) extract powder

**DOI:** 10.1002/fsn3.1949

**Published:** 2020-11-18

**Authors:** Edris Rahmati, Faroogh Sharifian, Mohammad Fattahi

**Affiliations:** ^1^ Department of Mechanical Engineering of Biosystems Urmia University Urmia Iran; ^2^ Department of Horticulture Urmia University Urmia Iran

**Keywords:** Moldavian balm, physicochemical properties, response surface methodology, spray‐drying

## Abstract

The present study was aimed to develop the powder from Moldavian balm extract using a spray dryer to preserve the valuable phytochemicals such as hydroxycinnamic acid and flavonoids. In order to produce optimum Moldavian balm spray‐dried powder, response surface methodology was applied. The inlet air temperature (120–180°C), compressed airflow rate (5–10 L/min), and carriers’ concentration (10%–30%) were kept as independent variables, while moisture content, drying performance, porosity, total phenol content, total flavonoid content, and antioxidant activity were selected as responses. The process was optimized with inlet air temperature of 140.36°C, compressed airflow rate of 9.13 L/min and carriers’ concentration of 18%, resulting in powder with moisture content of 7.68%, drying performance of 62.52%, porosity of 76.4%, total phenol content of 6.295 mg GAE/g, total flavonoid content of 0.378 mg QUE/g, and antioxidant activity of 51.78%. The optimized process led to attain the powder having significantly better phytochemical properties compared with others.

## Graphical abstract

A powder from Moldavian balm plant extract successfully obtained using spray‐drying. Air temperature, airflow, and carriers were experimental independent variables. Optimum conditions of variables were 140.36°C, 9.13 L/min, and 18%, respectively. Spray‐dried optimized powder retained significant amount of phytochemical compounds.

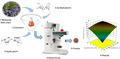

## 1 INTRODUCTION

In recent years, the use of medicinal plants has been increased dramatically with the development of human societies and the importance of herbal medicines. According to the World Health Organization, nearly 80% of the world's population still relies solely on medicinal plant products. The annual demand for medicinal plants is $14 billion, which is likely to increase to $5 trillion by 2050 (Tripathy et al., 2015).

Moldavian balm (*Dracocephalum moldavica* L.) is an annual herb with white and blue flowers and aromatic leaves belonging to the lamiaceae family, which reaches 80 cm in height (Hussein et al., 2006; Nikitina et al., 2008). This plant is native to Central Asia and is naturalized in Central and Eastern Europe (Dastmalchi, Dorman, Koşar, et al., 2007). In Iran, it is distributed in the northern and northwestern parts of the country, particularly in the Alborz Mountains and west Azerbaijan province (Dmitruk & Weryszko‐Chmielewska, 2010; Mafakheri et al., 2012). The west Azerbaijan province has the greatest amount of under cultivation areas in the country. In 2018, it was reported that, about 170 ha of lands in the province of west Azerbaijan have been allocated to the cultivation of Moldavian Balm, of which about 3,340 tons been harvested. Moldavian Balm is a rich reservoir of secondary metabolites for humans and is an important medicinal herb that used in traditional medicine for the treatment of gastric disorders, headache, liver and cardiovascular dysfunction (Dastmalchi, Dorman, Laakso, et al., 2007).

The extract of Moldavian balm plant is mainly used in the pharmaceutical, cosmetic and also as flavoring in food, beverage, confectionery, and other products (Dmitruk & Weryszko‐Chmielewska, 2010; Hussein et al., 2006). The most important compound of the aqueous extract of the plant is reported to be Rosmarinic acid, and antioxidant and anti‐Alzheimer properties are also attributed to Rosmarinic acid (Dastmalchi, Dorman, Laakso, et al., 2007). There are also many reports considering anticancer, anesthetic, and antimicrobial activity of the plant extract (Chachoyan & Oganesyan, 1996; Sultan et al., 2008) due to the polar compounds present in the aerial parts of the plant such as hydroxycinnamic acid (rosmarinic acids, ferrolic and caffeic acids) and flavonoids (apigenin, acacetin, quercetin, diosmetin, kaempferol, agastachioside, and salvigenin; Dastmalchi, Dorman, Laakso, et al., 2007; Yang et al., 2014). Recent studies have confirmed sedative (Dastmalchi, Dorman, Laakso, et al., 2007), anti‐aging (Maimaitiyiming et al., 2014), anti‐platelet, cardioprotective (Jiang et al., 2014), and neuroprotective (Sun et al., 2014) properties of the plant.

In order to optimize the valuable Moldavian balm extract, prevent compounds oxidation and quality alterations during storage as well as production in large scale, it is required to manufacture new processing methods with specific condition. Therefore, it is necessary to introduce a novel method for processing the extract to preserving the phytochemical compounds and access the dried extract of Moldavian balm. Several methods such as freeze‐drying, spray drying, and spouted‐bed drying have been used to dry of medicinal plant extracts (Cortés‐Rojas & Oliveira, 2012).

Spray dryer has been brought into spotlight due to the continuity of the process, potential capacity in converting a liquid to powder with preservation of properties (physicochemical stability), high flexibility, economical operation, and easy control of operating conditions (Desai & Jin Park, 2005; Hojjati et al., 2011; León‐Martínez et al., 2010; Oliveira & Petrovick, 2010). Spray drying is the process of converting liquid to the dried particles by spraying the liquid (solution, suspension, emulsion, or gel) into the dryer chamber, which provides sufficient volume of hot air to evaporate liquid droplets. In spray dryers, despite the high temperature used to drying, the particles sprayed due to the loss of moisture remain at a lower temperature and for a very short time, so the thermal damage to the product is reduced (Ameri & Maa, 2006; Fang & Bhandari, 2011; Yatsu et al., 2011).

Before utilizing spray dryer, a material named “carrier” or “drying matrix” should be added to the extract or suspensions in order to protect the bioactive components and improve the drying performance and quality of the final powder. The most prevalent kinds of carriers are carbohydrates, gums, cellulose derivatives, and synthetic polymers (Chiu et al., 2007; Ersus & Yurdagel, 2007; Tuyen et al., 2010). Due to the low cost, low viscosity, and good solubility, maltodextrin is one of the carriers that has been successfully utilized in spray‐drying process (Boonyai et al., 2004; Cano‐Chauca et al., 2005). Spray dryer has been successfully used to produce the powder of Guava fruit juice (Patil et al., 2014), Sisal liquids extracts (Salas et al., 2017), and *Murraya koenigii* leaves extract (Sablania & Bosco, 2018). The quality of the final powder and the drying performance depend on the drying operational conditions and the concentration of the carriers (Zuidam & Shimoni, 2010).

Therefore, in order to achieve a high quality powder and also to reduce costs, it is essential to select the optimal levels of each of the process variables. Response surface methodology (RSM) is widely applied to model and optimize operational parameters in various processes. The main advantage of the RSM is to reduce the number of necessary tests and then to create a mathematical relationship between independent variables and the response variable and to find the optimal levels of independent variables (Lee et al., 2006). So far, numerous studies have been carried out on Moldavian balm plant and identification of its phytochemical compounds. However, no investigation has been reported clearly and elaborately on the Moldavian balm drying process and the effect of spray‐drying conditions on the physicochemical properties of its powder.

Hence, the focus of this study gathers on describing the optimization process for development of Moldavian balm powder using spray dryer and maltodextrin and apple pectin as carriers. The dried Moldavian balm powder contributes to the stability of extract bioactive components, and also makes it easier to transport and storage, in addition to the possibility of achieving the unique medicinal properties of the plant throughout the year. Also, in order to predict each of the responses, regression models will be presented for different drying conditions. Eventually, the effect of each independent variable on the physicochemical properties of the powder is investigated.

## 2 MATERIALS AND METHODS

### 2.1 Raw materials

The aerial parts of Moldavian Balm plant (*D. moldavica* L.) were harvested after four months of planting from a farm in Urmia, Iran, at the full bloom flowering stage where 80% of the flowers appeared (in mid‐August). After the preparation of the plant samples (30 kg), the leaves of the plant isolated and then were dried in shade (24 ± 3°C). The moisture content of dried samples were 11% (wet basis).

### 2.2 Extract preparation

The dried Moldavian Balm plant was extracted using maceration method with ethanol 50% (v/v), plant to solvent ratio of 1/10 (w/v), and an extraction temperature of 25°C. After 48 hr, the extract was filtered using a filter paper. Then, the extract was concentrated in a rotary evaporator (Buchi Rotavapor R‐205, Switzerland) at 38 ± 2°C and 100 rpm to obtain concentrate of 4% (40 g/L). Afterwards, carriers were added to the extract in different levels (1:10, 2:10, 3:10 (g:g); apple pectin: Maltodextrins, with dextrose equivalent of 16). The carriers purchased from Sigma‐Aldrich. The phytochemical compounds of extract were measured before drying, and the following results were obtained: total phenol content: 6.45 mg GAE/g DW, total flavonoid content: 0.41 mg QUE/g DW and antioxidant activity: 60%.

### 2.3 Spray drying

**FIGURE 1:**
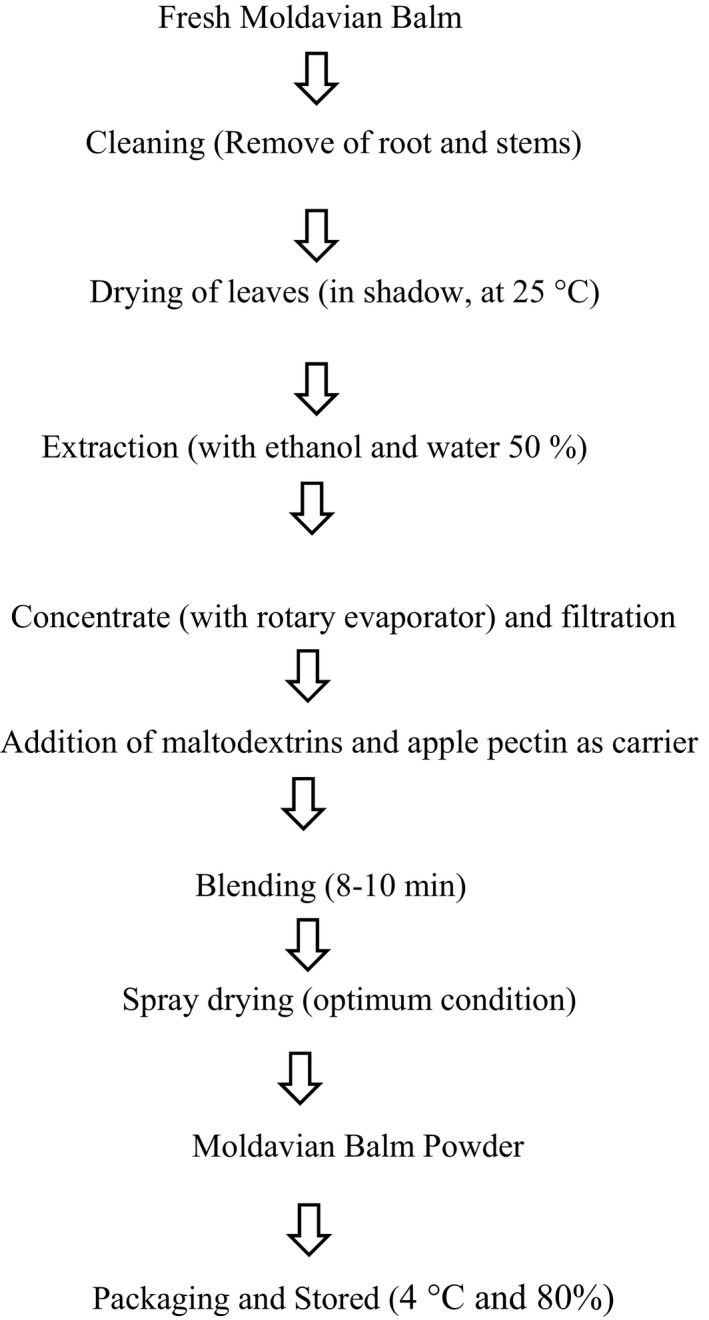
Process diagram for the development of Moldavian balm powder

The process of development of Moldavian balm powder has been described in Figure 1. The drying process of Moldavian balm plant extract was performed using a laboratory‐scale spray dryer Buchi B‐191 (Buchi) with specifications of: 220VAC‐14A‐3000W. The process of spraying the extract into a dryer chamber was carried out by two‐flow nozzle with 0.7 mm diameter under compressed air generated by compressor device. The ambient air enters the drying chamber after passing through a heating element in concurrent flow regime with the extract. Finally, the dried particles were collected at the bottom of the cyclone and separated from the air. In all experiments, the ambient air temperature and feed flow rate were steadied at 25°C and 16 ml/ min, respectively.

### 2.4 Experiment variables and optimization of the spray‐drying condition of Moldavian Balm powder

A 3^3^ Box‐Behnken design was followed to evaluate the effect of independent variable on the physicochemical properties of Moldavian Balm extract powder (Table 1). All experiments were evaluated at three different levels of inlet air temperature (120–180°C), compressed airflow rate (5–10 L/min), and carrier concentration (10%–30%). Moisture content, drying performance, porosity, total phenol content, total flavonoid content, and antioxidant activity were considered as the response variables due to its more effective role in evaluating the final Moldavian balm powder in comparison with other responses. In order to predict the response variables and the importance of each independent variable's effect, quadratic regression models were used as follows:

$$Y_{i}=\;a_{0}+a_{1}X_{1}+a_{2}X_{2}+a_{3}X_{3}+a_{11}X_{1}^{2}+a_{22}X_{2}^{2}+a_{33}X_{3}^{2}+a_{12}X_{12}+a_{13}X_{13}+a_{23}X_{23}$$

**TABLE 1:** Experimental design for response surface analysis of the effect of processing conditions on the quality of Moldavian balm powders

Run	Independent variables			Responses					
	Factor 1: Inlet temp (°C)	Factor 2: Airflow rate (L/min)	Factor 3: Carrier (g/g)	MC (%)	DP (%)	P (%)	TPC (mg GAE/g DW)	TFC (mgQUE/g DW)	AA (%)
1	120	5	0.2	11.3	16	84	4.36	0.23	32.56
2	150	10	0.1	7.8	65	74	5.31	0.26	36.5
3	150	7.5	0.2	7.9	61	85	6.4	0.4	58
4	180	7.5	0.3	6.36	69	90	4.5	0.23	34.11
5	150	7.5	0.2	7.9	59	85	6.4	0.4	58
6	120	10	0.2	8	55	64	5.24	0.3	36
7	180	10	0.2	6.2	73	87	3.75	0.21	22.09
8	180	5	0.2	7.86	65	90	5.42	0.28	21.42
9	150	7.5	0.2	7.9	60	85	6.4	0.4	58
10	150	10	0.3	6.84	68	80	4.7	0.26	34.72
11	150	5	0.3	10	38	90	5.06	0.24	35.33
12	120	7.5	0.3	9.5	40	75	3	0.2	42.41
13	120	7.5	0.1	10.3	30	74	5.83	0.32	43.21
14	150	5	0.1	10.1	35	88	5.35	0.27	39.98
15	180	7.5	0.1	7.66	67	88	3.8	0.21	28.29

Note

MC, DP, P, TPC, TFC, and AA denotes the moisture content, drying performance, porosity, total phenol content, total flavonoid content, and antioxidant activity, respectively. Number of replicates = 3.

In this equation, *Y* is the response variable; *X*_1_, *X*_2_, and *X*_3_ are independent variables; *a*_1_, *a*_2_, and *a*_3_ are linear regression coefficients; *a*_11_, *a*_22_, and *a*_33_ are quadratic regression coefficients; and *a*_12_, *a*_13_, and *a*_23_ are interaction regression coefficients. Design‐Expert software (Version 10) was used to analyze the data and also optimize the independent variables simultaneously, to achieve optimal responses.

### 2.5 Physicochemical analysis of Moldavian Balm powder

#### 2.5.1 Moisture content

The moisture content of powders was calculated by AOAC method (Horwitz, 2000). Two grams powder from each sample was taken into a preweighed petri dish. Then, the petri dish kept in vacuum oven at 70°C until it showed constant weight. The value of lost water from the samples was measured by a digital balance (DJ‐V320A, AND‐Japan) after 24 hr. Finally, the moisture content was calculated on wet basis.

#### 2.5.2 Drying performance

The performance of spray dryer is the determining factor of final powder amount. It was obtained from the ratio of amount dried powder to solid particles existing in the extract (Santhalakshmy et al., 2015).

#### 2.5.3 Porosity

The porosity of the powdered samples was measured using powders true density and bulk density as follows (Tonon et al., 2008).

$$\text{Porosity}=1‐\frac{\left(\text{bulk density}\right)}{\left(\text{particle density}\right)}$$

#### 2.5.4 Total phenol content

Total phenol content was measured by Folin‐Ciocalteu (FC) assay according to the method described by Marinova et al. (2005) with some modifications. A sample of 100 mg of powder was dissolved in 20 ml of ethanol solution at 50%, and then, 5 µl was withdrawn. Afterward, 180 µl of distilled water and 1,200 µl of FC reagent were added to the samples in test tubes. 12 ml of sodium carbonate (7.5%) was added after 5 min, the samples were kept at ambient temperature for 90 min, the absorbance of samples was determined by a Shimadzu UV2100 spectrophotometer at a wavelength of 760 nm, and the total phenol content was calculated and presented in mg GAE/g DW.

#### 2.5.5 Total flavonoid content

Total flavonoid content was calculated by the aluminum chloride colorimetry method and quercetin as the standard material (Beketov et al., 2005) with some modifications. 100 mg of powder was dissolved in 20 ml of ethanol solution at 50%, and then, 5 µl was withdrawn. Then, 300 µl of 10% aluminum chloride solution, 150 µl of sodium nitrite, and 1,000 µl of 1 mol/L NaOH acetate solution were added to the samples. Finally, the volume of this solution was increased to 5 ml by adding distilled water. The absorbance of samples was determined in the wavelength of 380 nm relative to the control treatment, and the amount of total flavonoids was calculated and presented in mg QUE/g DW.

#### 2.5.6 Antioxidants activity

In order to quantify the antioxidant activity, 100 mg of powder was dissolved in 20 ml of ethanol solution at 50% and 10 µl of it was taken into a test tube and mixed with 2,000 µl of DPPH solution. After that, the samples were kept at ambient temperature for 30 min and the absorbance was determined by spectrophotometer at the wavelength of 517 nm. Finally, the antioxidant activity was obtained according to the following equation (Akowuah et al., 2005):

$$\%\text{DPPH}=\frac{A_{\text{blank}}‐A_{\text{sample}}}{A_{\text{blank}}}$$

where *A*_blank_: blank absorption value; *A*_sample_: the sample's absorption value; and %DPPH is the inhibition percentage of free radicals.

## 3 RESULTS AND DISCUSSION

### 3.1 Optimization of experimental variables

In order to develop optimum Moldavian balm extract powder, the experiments were conducted to determine the high and low levels of independent variable such as inlet air temperature, compressed airflow rate, and carrier concentration. In lower inlet temperature (120°C), the drying performance was reduced. Similar results were obtained regarding low carrier concentration (10%) and low compressed airflow (5 L/min). With increasing inlet air temperature (>150°C) and compressed airflow rate (>7.7 L/min), the phytochemical compounds of dried extract decreased, while the physical properties of the samples were close to their optimal values. However, by increasing carrier concentration (>20%), the phytochemical compounds of the samples were further preserved.

Response surface methodology was employed to numerically optimize spray‐drying conditions and to achieve most desired characteristic of spray‐dried powder. The desired goal for each independent variables and responses is shown in Table 2. The maximum or minimum value of each response was selected based on maximum desirability function, and also, independent variables were retained in range. Figure S1 shows the optimal levels of independent variables and the predicted value for responses.

**TABLE 2:** Criteria and outputs of the numerical optimization of the responses for Moldavian balm powder

Name	Goal	Lower limit	Upper limit	Importance
Inlet air temperature (°C)	In range	120	180	3
Compressed air flow (L/min)	In range	5	10	3
Carrier concentration (%)	In range	0.1	0.3	3
Moisture content (%)	Minimize	6.2	11.3	3
Drying performance (%)	Maximize	16	73	3
Porosity (%)	Minimize	64	90	3
Total phenol content (mg GAE/g DW)	Maximize	3	6.4	3
Total flavonoid content (mg QUE/g DW)	Maximize	0.2	0.4	3
Antioxidant activity (%)	Maximize	21.42	58	3

The optimum conditions for spray drying of Moldavian balm extract were, inlet temperature (140.36°C), compressed airflow rate (9.13 L/min), and carrier concentration (18%) for obtaining desired characteristics of spray‐dried powder such as moisture content (7.68%), drying performance (62.52%), porosity (76.4%), total phenol content (6.295 mg GAE/g DW), total flavonoid content (0.378 mg QUE/g DW), and antioxidant activity (51.78%).

### 3.2 Regression models

To predict each of the responses, six coded regression models were obtained using the experimental data of Table 1. Only the statistically significant parameters were used to formulate the model. The quadratic regression models, which have been obtained by analyzing the response surface, reveal the effect of independent variables on moisture content, drying performance, porosity, total phenol content, total flavonoid content, and antioxidant activity (1–6). According to the regression coefficients, the air temperature had the maximum effect on the moisture content, drying performance, and porosity of the samples, while the carrier concentration showed the minimum impact on the parameters. Also, carriers were identified as the most effective factor in maintaining phenolic and total flavonoids compounds.

(1) $$\text{Moisture content}\left(\text{MC}\right)=+7.96‐1.38\times A‐1.30\times B‐0.40\times C+0.41\times \text{AB}+0.33\times B^{2}+0.44\times C^{2}$$

(2) $$\text{Drying performance}\;\left(\text{DP}\right)=+60+16.62\times A+13.37\times B+2.25\times C‐7.75\times \text{AB}‐3.87\times A^{2}‐3.87\times B^{2}‐4.63\times C^{2}$$

(3) $$\text{Porosity}\;\left(P\right)=+84.54+7.25\times A‐5.88\times B+1.38\times C+4.25\times \text{AB}‐2.36\times A^{2}$$

(4) $$\text{Total phenol content}\;\left(\text{TPC}\right)=+6.40‐0.38\times C‐0.64\times \text{AB}+0.88\times \text{AC}‐1.26\times A^{2}‐0.44\times B^{2}‐0.85C^{2}$$

(5) $$\text{Total flavonoid content}\;\left(\text{TFC}\right)=+0.40‐0.015\times A‐0.016\times C‐0.035\times \text{AB}+0.035\times \text{AC}‐0.081\times A^{2}‐0.064\times B^{2}‐0.079\times C^{2}$$

(6) $$\text{Antioxidants activity}\;\left(\text{AA}\right)=+58.00‐6.03\times A‐14.80\times A^{2}‐15.18\times B^{2}‐6.19\times C^{2}$$

where A = inlet air temperature (°C); B = airflow rate (L/min); C = carrier (g).

### 3.3 Effect of process variables on physicochemical properties of Moldavian balm powder

#### 3.3.1 Moisture content

**FIGURE 2:**
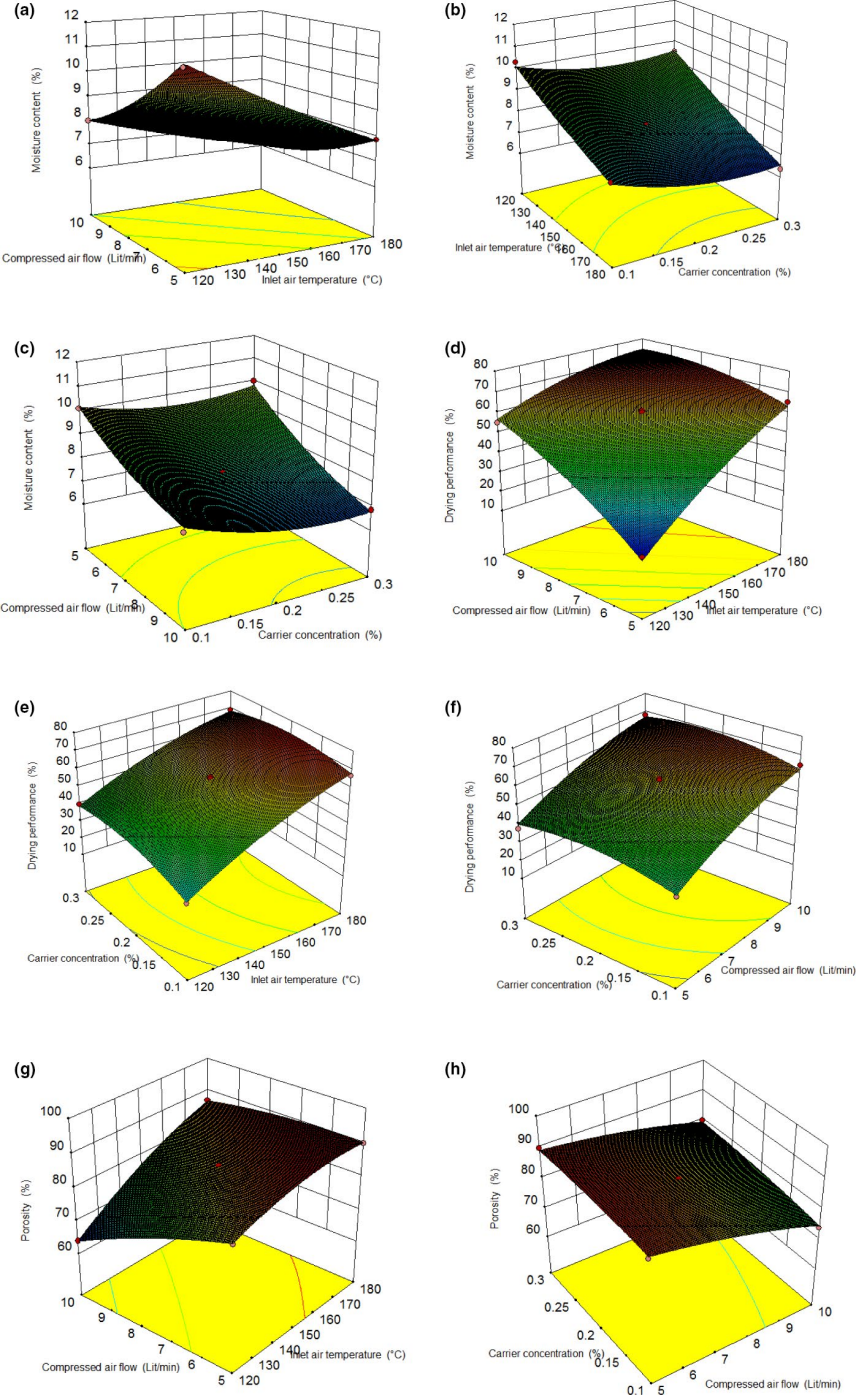
Response surface plots (3D) showing the effects of process parameters on: moisture content (a–c), drying performance (d–f), and porosity (g,h)

Moisture content is a key factor of the extract powder, which is relevant to the drying performance. Moisture content has a great impact on the quality parameters of powders such as flowability, stickiness, storage stability, caking, and oxidation reactions (Islam et al., 2017). Moisture content of Moldavian balm extract powder varied from 6.2% to 11.3% as shown in Table 1. The analysis of variance data for response variables with significant levels is presented in Table 3. All the independent variables showed significant effects on the moisture content (*p* < .01). Figure 2a–c shows the influence of independent variables on the spray‐drying moisture content. The fact that the moisture content decreases with the increase in inlet air temperature may be due to higher temperature gradient between atomized particles and the hot air that results in the greater particle's heat transfer rate and consequently the increase of the evaporation rate. Hu et al. (2016) and Patil et al. (2014) reported similar results during the microencapsulation of guava powder and *Brucea javanica* oil, respectively. These findings, however, are not in line with the observation by (Santhalakshmy et al., 2015) for spray‐dried Jamun fruit juice. Increase in compressed airflow rate showed a negative effect on moisture content; similar results were observed for moisture content of tomato pulp powder (Goula & Adamopoulos, 2005).

**TABLE 3:** Significant levels of Moldavian Balm powder responses using RSM

Source (P > F)	Responses					
	MC (%)	P (%)	DE (%)	TPC (mg GAE/g DW)	TFC (mg QUE/g DW)	AA (%)
Model	<0.0001	<0.0001	<0.0001	0.0004	0.0003	0.0003
*X*_1_	<0.0001	<0.0001	<0.0001	0.1581	0.0214	0.0006
*X*_2_	<0.0001	<0.0001	<0.0001	0.0949	0.7941	0.9976
*X*_3_	0.0026	0.0048	0.0427	0.0034	0.0159	0.8331
*X*_1_ *X*_2_	0.0096	0.0001	0.0012	0.0016	0.0028	0.5644
*X*_1_ *X*_3_	0.2696	0.5623	0.1503	0.0003	0.0028	0.2005
*X*_2_ *X*_3_	0.0859	0.0558	1.0000	0.4697	0.2956	0.5509
$X_{1}^{2}$	0.3625	0.0019	0.0251	<0.0001	<0.0001	<0.0001
$X_{2}^{2}$	0.0241	0.0308	0.0251	0.0089	0.0002	<0.0001
$X_{3}^{2}$	0.0078	0.1339	0.0130	0.0005	<0.0001	0.0032
Lack of fit	—	—	0.1061	—	—	—
*R* ^2^	.9937	.996	.9933	.9858	.9880	.987
CV	2.41	0.98	4.41	4.06	4.58	5.8

Note

*X*_1_, *X*_2_, *X*_3_, and CV denotes the inlet air temperature (°C), compressed airflow (L/min), carrier concentration (%), and coefficient of variation, respectively.

Particle size reduction, as a result of higher airflow rate, increased particles surface area and its ratio to volume. Therefore, this property assist to increase partially loss of moisture as a result of faster diffusion rate. So residual moisture content of dried extract decreased correspondingly. Also, moisture content decreased as a result of apple pectin enhancement up to 25% but the moisture content increased at higher percentage (Figure 2a–c). The initial decrease in moisture content of the powder samples may be due to increase in feed solids and a reduction in total moisture for evaporation (Fazaeli et al., 2012). Similar results were reported by Patil et al. (2014) in spray drying of fruit juice guava. However, the concentration of apple pectin (>25%) showed a positive effect on moisture content of powder samples.

At the high concentration of pectin (>25%), the feed viscosity increased that leads to increase in the particle size during the spraying step, and therefore, the amount of residual moisture in the dry particles increased.

#### 3.3.2 Drying performance

Drying performance plays a crucial role in producing any spray‐dried powder. The drying performance of the spray‐dried extract was in the range of 16%–73% (Table 1). Figure 2d–f shows the effect of independent variables on the spray‐dried powder's performance. The increment of inlet air temperature led to the increase of Moldavian balm powder samples’ performance, which may be due to the greater efficacy of heat and mass transfer process occurred at higher temperatures. This result is consistent with previous studies (Muzaffar & Kumar, 2015; Sablania & Bosco, 2018), studying *M. koenigii* and tamarind pulp at various inlet air temperature. The increase of compressed airflow rate caused the increment of drying performance of Moldavian balm powder samples (Figure 2d–f). Higher compressed airflow rate led to decrease in particles’ diameters. The smaller particles are denser and also have lower moisture content, therefore, the probability of particles’ stickiness to each other and also to chamber's wall decreases which finally results in increasing drying performance (Largo Avila et al., 2015). Increase in concentration of apple pectin in the liquid extract up to 25% leads to the increase of drying performance and then decreased at higher apple pectin concentration. The initial increase in drying performance of the powders due to role of pectin as a stabilizer and thickening agent leading to create a thin nonsticky coat around the particles during the drying process. Higher concentration of apple pectin (>25%), however, showed a negative effect on drying performance of powder samples, which can be due to the increase in mixture viscosity, that subsequently results in producing a sticky substance in the drying chamber. Thus, time taken for particle formation increases and then higher amounts of moisture will be remained in particles.

Previously, it has been reported that increase in maltodextrin concentration resulted in reduction in process yield of the powder during spray drying of açai (*Euterpe oleracea Mart*; Tonon et al., 2008).

#### 3.3.3 Porosity

Porosity is considered as one of the most important characteristics of powders because the powder samples are very prone to oxidation. The large value of porosity shows a large number of spaces between particles encompassing oxygen for degradation. Also, low porosity powders are of smaller chance for degradation (Islam et al., 2017; Santhalakshmy et al., 2015). The porosity value of the Moldavian balm extract powders oscillates within the range of 64%–90% (Table 1). The effect of process variables on porosity of the powder samples is shown in Figure 2g,h. The porosity of powder samples increased with the decline in bulk density which may be due to the increment in inlet air temperature. It can be stated that the evaporation rate increased at higher temperatures, so the particles were formed more rapidly, and then, there was not enough time for shrinking and decreasing the size of particles. However, the compressed airflow rate showed a negative effect on powder porosity. With increase in compressed airflow rate, particle size and space between them decreased and so porosity of powder samples declined. Similar result were reported by Jafari et al. (2017) during spray drying of pomegranate juice powder. The increment of carrier concentration led to the increment of porosity in powder samples. By increasing the carrier concentration, the moisture content of powder samples decreased. As a result, powder with low moisture content is of a lower bulk density and therefore of higher porosity (Chegini & Ghobadian, 2005).

#### 3.3.4 Total phenol content

**FIGURE 3:**
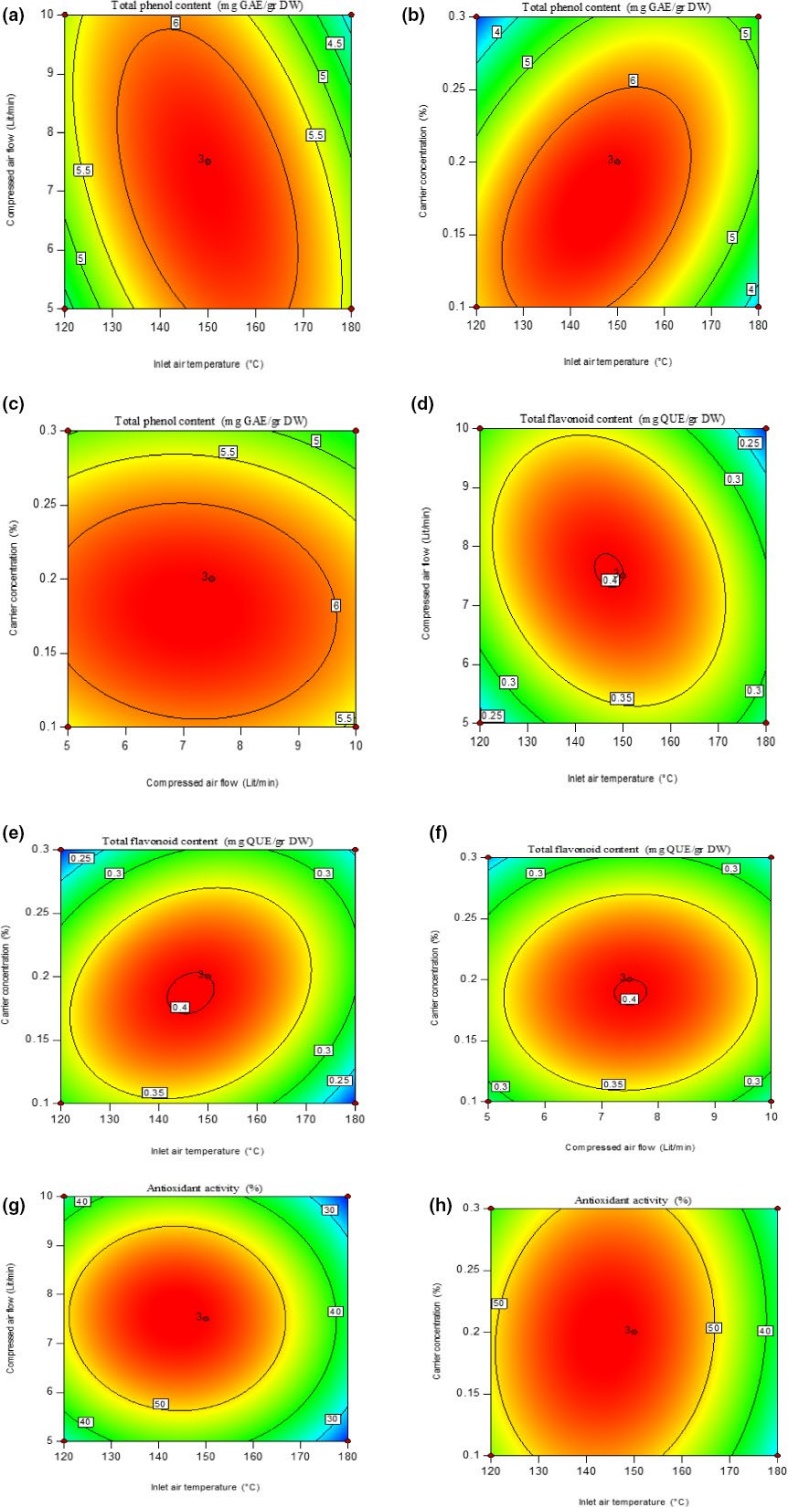
Contour plots for total phenol content (mg GAE/g DW) (a–c), total flavonoid content (QUE/g DE) (d–f), and antioxidant activity (g,h) of the powders produced by spray‐drying Moldavian balm extract

The total phenol content of spry‐dried extract varied between 3.0 and 6.4 mg GAE/g DW (Table 1). Total phenol content was drastically influenced by carrier concentration as shown in Table 3. Increase in carrier concentration showed positive effect on total phenol content (Figure 3a–c). On the other hand, total phenol content and bioactive compound were less affected by temperature because of protective effect of pectin as a polymeric coat for heat‐sensitive components (Li et al., 2008; Suhag & Nanda, 2016). The double effects of air temperature and compressed airflow rate variables showed significant effects on the total phenol content (*p* < .01) Table 3. Figure 3a–c shows that, the higher air flow rate caused a decrease in total phenol content. With increase in air flow rate, the size of particles decreases, so the thermal damage to particles would increase. This in turn, leads to decrease in total phenol content. However, increase in inlet air temperature up to 150°C had positive effect on total phenol content, while at higher temperature TPC value significantly decreased. It can be concluded that, high temperatures have led to changes in powder nature and degradation of phenolic compounds; for the reason that phenols are heat‐sensitive compounds (Podsędek, 2007). Sablania and Bosco (2018) reported the same results for spray drying of *M. koenigii* leaves extract at various inlet air temperature.

#### 3.3.5 Total flavonoid content

The levels of total flavonoid content of Moldavian Balm powdered samples ranged from 0.2 to 0.4 mg QUE/g DW (Table 1). The ANOVA of Moldavian Balm powdered samples showed that the simple effects of carriers and inlet air temperature variables had a great influence on total flavonoid content at a significant level of 1% and 5%, respectively, Table 3. Increase in inlet air temperature and carrier concentration showed a significantly decrease in total flavonoid content (Figure 3d–f). At the high temperatures, despite protective effect of carriers, the total phenol content decreased. These results were in complete accordance with the data presented by (Couto et al., 2012) during spry‐drying of rosemary extract. Also, Souza et al. (2008) observed similar results when rosemary extract was dried using spray and spouted‐bed dryers. According to these authors, oxidative condensation or decomposition of thermolabile compounds and also the possibility of losses of essential oil and other volatile substances during drying process may be among factors leading to the phenolic compound degradation. Total flavonoid content of Moldavian Balm powdered samples did not show significant effect with change in compressed airflow rate.

#### 3.3.6 Antioxidants activity

In this study, the antioxidant activity of the dried powder varied between 21.42% and 58% (Table 1). Antioxidant activity was significantly influenced by inlet air temperature as shown in Table 3. Increase in inlet air temperature showed significantly decrease in antioxidant activity due to the degradation of polyphenolic compounds and oxidative reactions occurring at higher temperature Figure 3g,h. Similar result for antioxidant activity was reported by Krishnaiah et al. (2015) for spray drying of *Morinda citrifolia* L. fruit extract. Other influencing factors are shown in Table 3.

## 4 OPTIMIZED SPRAY‐DRIED MOLDAVIAN BALM POWDER

In this study, independent factors for the development of Moldavian balm extract powder were optimized to minimize the moisture content, porosity, and maximize the drying performance, total phenol content, antioxidant activity, and total flavonoid content. The optimum independent factors with desired characteristic of Moldavian balm extract powder were obtained using a spray dryer. The inlet air temperature of 140.36°C, the compressed air flow rate of 13.9 L/min, and the carrier concentration of 18% led to the optimal response with the desirability function of 0.776. Moisture content, drying performance, porosity, total phenol content, total flavonoid content, and antioxidant activity in the final optimized powder were as follows: 7.68%, 62.52%, 76.4%, 6.3 mg GAE/g, 38/0 mg QUE/g DW, and 51.79%, respectively (Figure S1). The optimized inlet air temperature, compressed air flow rate, and carrier concentration were examined to evaluate the efficiency of regression models for forecasting and optimizing responses (Table 4). These results show validity of developed models for predicting and optimizing responses.

**TABLE 4:** Experimental and predicted values

Responses	Experimental value	Predicted value	|Relative error (%)|
Moisture content (%)	6.981	7.6803	10.02
Drying performance (%)	65.691	62.518	4.83
Porosity (%)	80	76.403	4.5
Total phenol content (mg GAE/g DW)	5.851	6.296	7.6
Total flavonoid content (QUE/g DW)	0.3394	0.3789	11.63
Antioxidant activity (%)	48.667	51.7809	6.4

## 5 CONCLUSIONS

In the present study, 15 different experimental runs were carried out using Box‐Behnken design for the purpose of investigating the physicochemical properties of Moldavian balm extract powder in different levels of inlet air temperature, compressed airflow rate and carrier concentration. The overall results are as follows:

(a) The increase of inlet air temperature led to decrease of moisture content, total phenol content, total flavonoid, and antioxidant activity, while, porosity, and drying performance had an increasing trend. Despite the positive effect of increasing temperature on moisture content and drying performance, other powder characteristics which were sensitive to high temperatures, changed correspondingly; (b) the increment of carrier concentration led to the decrease of moisture content, while porosity, total phenol content, and antioxidant activity had an increasing trend. In other words, the increment of carriers had a positive effect on chemical properties of the powder; (c) increase in compressed airflow rate resulted in the decrease of moisture content and porosity. Meanwhile, drying performance values increased correspondingly; (d) the inlet air temperature and compressed airflow rate variables had the most significant effect on moisture content, porosity, and drying performance. In addition, the carrier concentration had the most considerable influence on chemical properties of the powder.

Considering the abundant advantages of the Moldavian balm extract powder in various industries such as pharmaceutical and food, in order to develop optimum Moldavian balm powder, the inlet air temperature of 140.36°C, compressed air flow rate of 9.13 L/min, and carrier concentration of 18% were proposed as desirable conditions for simultaneous optimization of responses.

## CONFLICT OF INTEREST

The authors declare that they have no conflict of interest.

## ETHICAL APPROVAL

This study did not involve any human or animal testing.

## Supporting information

Fig S1

## Data Availability

The data that support the findings of this study are available in the supplementary material of this article.
