# Supplementary material for: Process optimization of spray‐dried Moldavian balm (Dracocephalum moldavica L.) extract powder
Source: Food Sci Nutr. 2020 Nov 18;8(12):6580–91. doi: 10.1002/fsn3.1949 (PMC7723224; doi:10.1002/fsn3.1949)
Supplement: Supplementary file 1 — Fig S1 [file FSN3-8-6580-s001.docx]

**Supplementary File**

**Process optimization of spray-dried Moldavian balm (*Dracocephalum moldavica L.*) extract powder**

Edris Rahmati^[[1]](#footnote-1)^, Faroogh Sharifian^1*^, Mohammad Fattahi^[[2]](#footnote-2)^

^*^ Corresponding author, E-mail address: f.sharifian@urmia.ac.ir


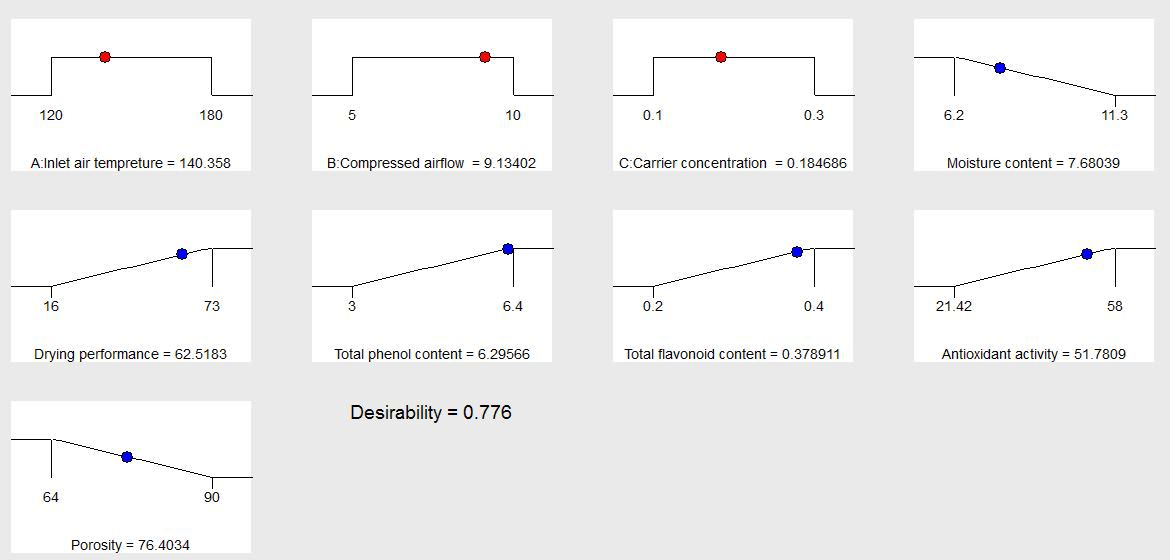
**Fig S1.**

1. - Department of Mechanical Engineering of Biosystems, Urmia University, Urmia, Iran. [↑](#footnote-ref-1)
2. - Department of Horticulture, Urmia University, Urmia, Iran. [↑](#footnote-ref-2)
